# The influence of propofol-based total intravenous anesthesia on postoperative outcomes in end-stage renal disease patients: A retrospective observation study

**DOI:** 10.1371/journal.pone.0254014

**Published:** 2021-07-22

**Authors:** Ho Bum Cho, Mun Gyu Kim, Sun Young Park, Sanghoon Song, Youn Sil Jang, Suyeon Park, Hyun Keun Lee, Jae Hwa Yoo, Ji Won Chung, Sang Ho Kim

**Affiliations:** 1 Department of Anesthesiology and Pain Medicine, Soonchunhyang University Hospital Seoul, Seoul, Republic of Korea; 2 Department of Biostatistics, Soonchunhyang University College of Medicine, Seoul, Republic of Korea; Scuola Superiore Sant’Anna, ITALY

## Abstract

**Background:**

To determine whether the anesthetic method of propofol total intravenous anesthesia (TIVA) is associated with postoperative outcome in ESRD patients, we evaluated the incidence of postoperative major adverse cardiac events (MACE), comparing propofol TIVA versus anesthesia with volatile anesthesia in ESRD patients.

**Methods:**

Retrospectively, we identified cases with ESRD patients who underwent surgery under general anesthesia. Patients were divided into those who received only volatile anesthesia (volatile group) and those who received only propofol TIVA (TIVA group). The incidence of MACE and potential confounding variables were compared separately in a univariate logistic model and subsequently by multivariate logistic regression.

**Results:**

Among the 2576 cases in ESRD patients, 1374 were in the TIVA group and 1202 were in the volatile group. The multivariate analysis included 12 factors, including the anesthesia method, of which five factors were significant. Factors that were associated with a significantly lower MACE risk included preoperative chloride concentration (OR: 0.96; 95% CI, 0.92–0.99), baseline SBP (OR: 0.98; 95% CI, 0.98–0.99), and propofol TIVA (OR: 0.37; 95% CI, 0.22–0.60).

**Conclusions:**

We inferred that the anesthetic method associated with the postoperative outcome in patients with ESRD.

## Introduction

End-stage renal disease (ESRD) is the last stage of chronic kidney disease requiring dialysis. The aging of the world’s population and improved survival in the ESRD population has resulted in a gradual increase in the prevalence of ESRD [1, 2]. As many as 9.7 million patients worldwide required dialysis in 2010 and the number is projected to double by 2030 [3]. The number of patients with ESRD who need surgery has also increased [4, 5]. Chronic kidney disease is an independent risk factor for postoperative death and cardiovascular events after noncardiac surgery [6]. Most ESRD patients have multiple comorbidities. Therefore, ESRD patients are more susceptible to the adverse effects of surgery and anesthesia [6–8]. As such, careful perioperative management and optimization of the anesthetic technique is important. It is increasingly recognized that the anesthetic technique and other perioperative factors have the potential to affect the surgical outcome [9].

Propofol is a suitable agent to induce and maintain general anesthesia because of its rapid onset, clear recovery, and several other properties that potentially affect the surgical outcome. However, there are conflicting findings about postoperative outcomes associated with intraoperative volatile anesthetics *versus* propofol. It has been reported that the cardioprotective effects of volatile anesthetics was superior to propofol by improving postischemic recovery in cardiac surgery with cardiopulmonary bypass [10]. On the other hand, many studies have found that propofol-based total intravenous anesthesia (TIVA), compared with volatile anesthetics, is associated with improved survival after surgery in cancer patients or patients without solid cancer [11, 12]. Propofol has antioxidant properties, as well as anti-inflammation and immunomodulatory effects that are organ protective and may contribute to a better surgical outcome compared with volatile anesthesia [13–19]. These findings suggest that the use of propofol may be beneficial in high-risk populations, such as ESRD patients. However, the prognostic significance of propofol TIVA in ESRD patients is uncertain.

While there are potential advantages, some clinicians may fear side effects. For example, propofol induces vasodilation and hypotension, so hemodynamic fluctuations could be more prominent during general anesthesia using propofol [20, 21]. Additionally, there is a concerns of overdose in ESRD patients, because most pharmacokinetic models used for TIVA were developed in young, healthy individuals.

We performed a retrospective database study to assess the relationship between anesthetic method and postoperative major adverse cardiac events (MACE), which are a major cause of morbidity and mortality after surgery, by comparing propofol TIVA and anesthesia with volatile anesthetics in ESRD patients after non-cardiac surgery. Variables associated with blood pressure fluctuations during general anesthesia were also evaluated.

## Materials and methods

### Study design

This retrospective cohort study was approved by the Soonchunhyang University Hospital Institutional Review Board (SCHUH 2020–09–001). Written informed consent was waived because of the retrospective nature of the study. We present the findings following the format recommended by the Strengthening the Reporting of Observational Studies in Epidemiology (STROBE) guidelines.

After approval from the institutional review board, all ESRD on hemodialysis patients in our institutional database (age 20–80 years) who underwent general anesthesia between March 2018 and April 2020 were initially included. Considering the high mortality related to the procedures, any patients who had undergone cardiac surgery, cancer surgery, organ transplantation, or emergency surgery were excluded. General anesthesia was not combined with any type of regional block. To clearly distinguish the effects of each anesthetic method, patients who received both forms of anesthesia within the study period, either during the same surgical procedure or for additional procedures, were excluded. Patients who had multiple procedures during the study period and received the same form of anesthesia remained eligible. Patients were divided into those who received only volatile anesthesia (volatile group) and those who received only propofol TIVA (TIVA group).

### Anesthetic management

We have a general anesthetic protocol for ESRD patients. Patients receive hemodialysis one or two days before surgery. And the laboratory tests including hemoglobin, platelet count, electrolytes, blood urea nitrogen, creatinine, albumin, phosphate, and calcium levels are checked after hemodialysis. Clinical patient characteristics such as underlying disease and the laboratory test results are formally recorded in the pre-anesthetic evaluation report form. Patients maintain cardio-protective medications like beta-blockers, anti-hypercholesterolemic agent, anti-arrhythmic medications, except anticoagulation therapy. Angiotensin receptor blocker or angiotensin converting enzyme inhibitors are discontinued on the day of surgery. When patients receive TIVA, the target concentrations of propofol and remifentanil are maintained at 2–5 μg/ml and 0–6 ng/ml, respectively, according to a bispectral index (BIS) value of 40–60. When patients receive volatile anesthesia, induction is performed using intravenous 1–1.5 mg/kg propofol. Anesthesia is maintained with the volatile anesthetics desflurane or sevoflurane. The volatile anesthetic dose is adjusted to achieve a target BIS value of 40–60. Remifentanil is maintained at 0–6 ng/ml as needed. Fentanyl (0.3–0.5 μg/kg) was injected at the beginning of skin closure, in both anesthetic methods.

As per our protocol, blood pressure and heart rate were measured every minute and recorded automatically. Patients received intravenous 4 mg ephedrine or 50 μg phenylephrine as necessary to maintain systolic blood pressure within 20% of the baseline, a pre-anesthetic value, during the operation. If these drugs were insufficient to maintain blood pressure and the vital signs were severely unstable, inotropic agents, including dopamine, dobutamine, epinephrine, or norepinephrine were infused continuously.

### Variables

Preoperative morbidities, including hypertension, atrial fibrillation, myocardial infarction (MI), current angina, congestive heart failure, valvular heart disease, asthma, chronic obstructive pulmonary disease (COPD), interstitial lung disease, diabetes mellitus, cerebrovascular accident, dementia, liver cirrhosis, and the American Society of Anesthesiologists rating scale were assessed based on the preoperative anesthetic record. Preoperative test results, including left ventricular ejection fraction by echocardiography and pulmonary function tests, as well as hemoglobin, platelet count, electrolytes, blood urea nitrogen, creatinine, albumin, phosphate, and calcium levels were recorded for analysis. Intraoperative records, including anesthetics, blood pressure, heart rate, BIS, SpO_2_, body temperature, the volume of blood loss, crystalloids or colloids infused, transfusion, anesthetic duration, and vasoactive agents used to stabilize vital signs were evaluated. We calculated the total duration when systolic blood pressure (SBP) was ≥ 140 mmHg or diastolic blood pressure (DBP) was ≥ 90 mmHg, defined as hypertensive duration, and calculated the duration of SBP ≤ 90 or mean blood pressure (MBP) ≤ 65 as hypotensive duration. A heart rate (HR) > 100 was considered tachycardia and < 50 as bradycardia. These variables were chosen as potential confounders, as they have either been shown or posited to affect outcomes [22–24].

### Data sources/Measurement

All data related to the procedure and anesthesia were obtained from electronic medical patient records and the real-time vital recorder file [25]. Data related to outcomes were obtained by submitting a batch data request to the Korean National Statistical Office (Microdata Integrated Service, on-demand, https://mdis.kostat.go.kr) and the electronic medical patient records. Since the Republic of Korea manages the death data of its citizens through the resident registration number, we can get correct data. And we extracted other postoperative MACEs from electronic medical records by requesting patients who newly diagnosed or managed within 30 days after surgery, using all related Insurance fee code, diagnostic code, prescription code. Surgical severity was graded from low to high according to the American College of Cardiology/American Heart Association guidelines [26].

### Statistical analysis

The primary outcome was postoperative MACE. Postoperative MACE was defined as a composite of all-cause death, sudden cardiac arrest, congestive heart failure, non-fatal MI, stress-induced cardiomyopathy, and new onset of cardiac arrhythmia, severe hypotension requiring inotropic support, new-onset pulmonary edema, pulmonary hypertension, cerebrovascular accident, and pulmonary embolism within 30 days after surgery. Patient demographics, comorbidities, test results, and intraoperative recordings were compared between the groups using the *t-*test or the Mann–Whitney *U-*test for continuous variables or the chi-square or Fisher’s exact test as appropriate. The Shapiro–Wilk test was used to assess the normality of the distribution. The overall incidence of MACE and potential confounding variables were compared separately in a univariate logistic model and subsequently by multivariate logistic regression. Variables with a p-value < 0.2 in the univariate analysis were used as candidate risk factors in the multivariate model. All variables included in the multivariate model should have complete data. Cases with missing co-variate data would excluded during the analysis. We used a two-sided 5% α-level to evaluate differences in the model. The results are expressed using the odds ratio (OR) and 95% confidence interval (CI) and plotted using a forest plot. All statistical analyses were performed using R software (version 4.0.2; R Foundation for Statistical Computing, Vienna, Austria).

## Results

**Fig 1:**
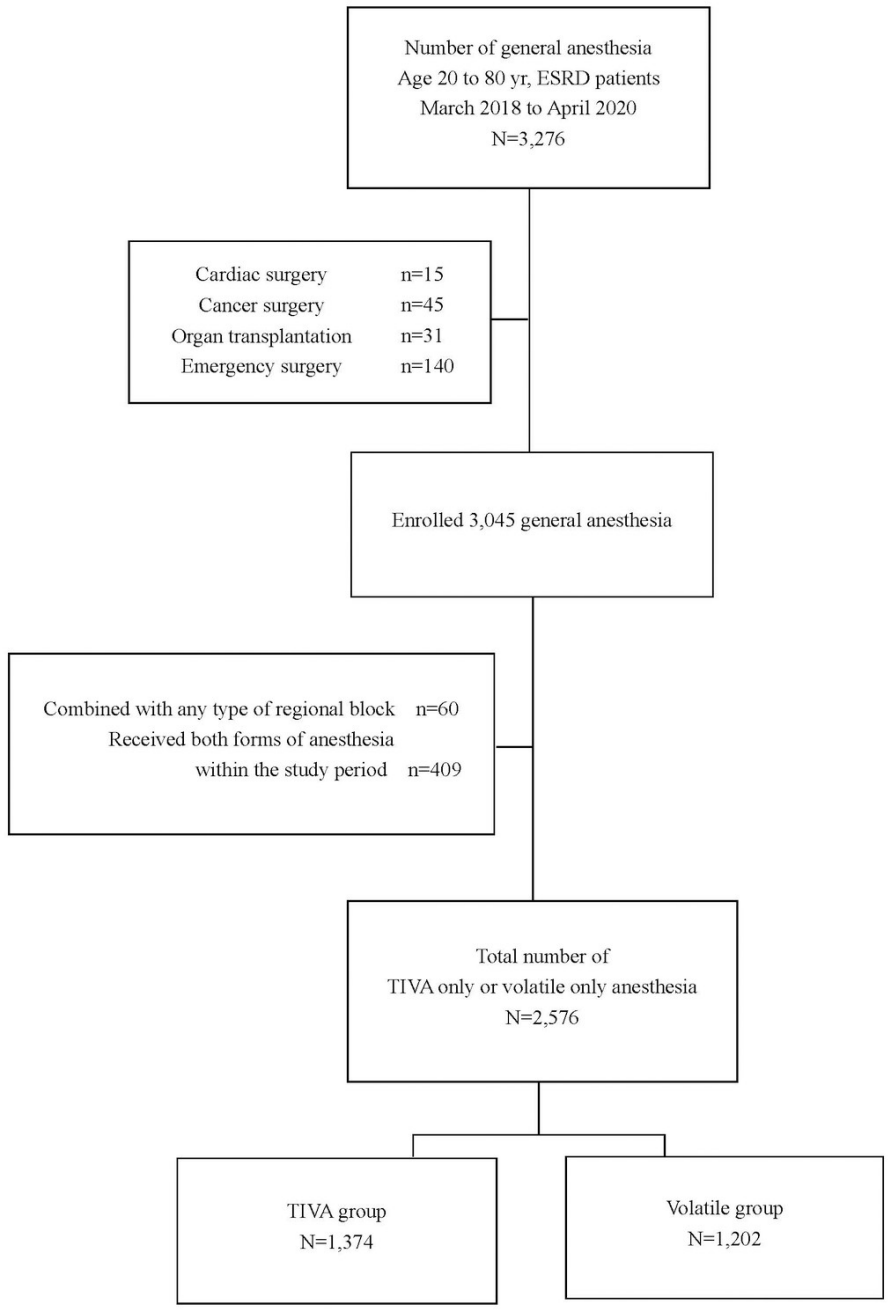
Study flow chart. TIVA group, patients who received only propofol-based total intravenous anesthesia; Volatile group, patients who received only volatile anesthesia.

In total, 3276 ESRD patients aged 20–80 years underwent general anesthesia during the study period. After the exclusion criteria were applied, 1374 patients remained in the TIVA group and 1202 were in the volatile group, and all were included for the analysis (Fig 1). Among the patients, 23.3% had multiple procedures during the study period (21.9% in TIVA group, 14.1% in volatile group).

Table 1 lists the clinical characteristics of patients, including preoperative morbidities and laboratory data. Gender, age, weight, body mass index, and the prevalence of several preoperative morbidities differed significantly between the two groups. Among the preoperative test results, forced expiratory volume in 1 sec (FEV1) (%) differed significantly but forced vital capacity (FVC) (%) and FEV1/FVC (%) did not differ significantly and had no clinical implication. Among preoperative laboratory test results, chloride concentration differed significantly between the groups. Surgery type and severity were similar between the two groups (S1 Table). Table 2 lists the intraoperative data, including anesthetics and vital signs. The baseline SBP, a pre-anesthetic value, was higher, and the baseline HR was lower in the TIVA group than in the volatile group. The mean MBP, DBP, and HR values during anesthesia were significantly lower in the TIVA group than in the volatile group. The calculated hypertensive duration, hypotensive duration, and tachycardia duration were significantly longer in the volatile group than in the TIVA group. The BIS and SpO_2_ values were significantly different but there was no clinical implication. The volume of blood loss and intraoperative crystalloid infusion was greater and anesthetic duration was longer in the volatile group than in the TIVA group. No transfusions occurred among the cases analyzed. Table 2 also lists data regarding intraoperatively administered vasoactive agents; no significant differences were observed between the two groups. The incidence of postoperative MACE was significantly lower in the TIVA group than the volatile group (P < 0.001) but the cause of the category distribution did not differ significantly between the two groups (Table 3). No pulmonary hypertension or cerebrovascular accident was detected within 30 days after surgery.

**Table 1:** Clinical patient characteristics.

Characteristics	Total (n = 2576)	TIVA group (n = 1374)	Volatile group (n = 1202)	P-value**
**Demographic data**				
**Gender (male: female)**	1375 (53%): 1201 (47%)	774 (56%): 600 (44%)	601 (50%): 601 (50%)	0.002^#^
**Age**	64.26 ± 13.31	65.63 ± 12.79	62.69 ± 13.71	<0.001^#^
**Weight**	60.59 ± 12.37	61.06 ± 12.06	60.05 ± 12.69	0.039^#^
**Height**	160.91 ± 9.86	160.89 ± 10.02	160.92 ± 9.67	0.946
**Body mass index**	23.31 ± 4.43	23.53 ± 4.63	23.06 ± 4.17	0.007^#^
**Comorbidities**				
**Hypertension**	2117 (82%)	1169 (85%)	948 (78%)	<0.001^#^
**Atrial fibrillation**	164 (6%)	76 (6%)	88 (7%)	0.076
**Previous MI**	91 (4%)	36 (3%)	55 (5%)	0.01^#^
**Current angina**	188 (7%)	76 (6%)	88 (7%)	0.566
**Congestive heart failure**	84 (3%)	46 (3%)	38 (3%)	0.877
**Valvular heart disease**	338 (13%)	153 (11%)	185 (15%)	0.002^#^
**Asthma**	43 (2%)	22 (2%)	21 (2%)	0.893
**COPD**	30 (1%)	15 (1%)	15 (1%)	0.854
**Interstitial lung Disease**	1 (0%)	0 (0%)	1 (0%)	0.467
**Diabetes mellitus**	1303 (51%)	756 (55%)	547 (46%)	<0.001^#^
**Cerebrovascular accident**	382 (15%)	180 (13%)	202 (17%)	0.010^#^
**Dementia**	55 (2%)	24 (2%)	31 (3%)	0.186
**Liver cirrhosis**	51 (2%)	25 (2%)	26 (2%)	0.629
**Pre-anesthesia evaluation**				
**ASA**				0.109
**3**	2204 (86%)	1329 (97%)	1147 (95%)	
**4**	100 (4%)	45 (3%)	55 (5%)	
**Left ventricular ejection fraction (%)**	61.2 ± 7.69 (n = 1453)	61.3± 7.27 (n = 700)	61.2 ± 8.14 (n = 753)	0.743
**Pulmonary functions test**				
**FVC (%)**	91.4 ± 15.33	91.7 ± 14.86	91.2 ± 15.86	0.438
**FEV**_**1**_ **(%)**	93.5 ± 23.1	94.3 ± 28.39	92.5 ± 14.87	0.035^#^
**FEV**_**1**_**/ FVC (%)**	73.2 ± 33.34	73.9 ± 40.68	72.3 ± 22.13	0.206
**Preop. Hemoglobin (g/dL)**	11.1 (10, 12.1)	11.1 (10, 12)	11.1 (10, 12.1)	0.058
**Preop. platelet count (10**^**9**^**/L)**	185 (142, 230)	183 (140, 226)	189 (144, 237)	0.159
**Preop. Sodium (mmol/L)**	140.1 ± 8.79	140.2 ± 10.83	139.4 ± 5.59	0.239
**Preop. Potassium (mmol/L)**	4.5 ± 0.73	4.4 ± 0.76	4.5 ± 0.69	0.421
**Preop. Chloride (mmol/L)**	100.5 ± 4.47	100.7 ± 4.74	100.3 ± 4.12	0.021^#^
**Preop. Blood urea nitrogen (mg/dL)**	35.8 ± 27.25	35.2 ± 26.18	36.3 ± 28.35	0.289
**Preop. Creatinine (mg/dL)**	5.6 ± 3.47	5.6 ± 3.37	5.5 ± 3.58	0.212
**Preop. Albumin (g/dL)**	4.2 ± 0.99	4.2 ± 0.64	4.1 ± 1.28	0.073
**Preop. Phosphate (mg/dL)**	4.4 ± 1.74	4.5 ± 1.78	4.4 ± 1.69	0.140
**Preop. Calcium (mg/dL)**	9.3 ± 0.94	9.3 ± 0.89	9.3 ± 1	0.176

*Abbreviation: MI, myocardial infarction; COPD, chronic obstructive pulmonary disease; ASA, American Society of Anesthesiologists; FVC, forced vital capacity; FEV1, Forced Expiratory Volume in One Second; Preop. Preoperative.

**P-value for an analysis between TIVA group and volatile group

***All continuous variables are reported as mean ± SD or median (IQR) and all categorical variables as n (proportion, %). Data were analyzed using t-tests or Mann-Whitney U test for continuous variable, chi-square or Fisher’s exact test as appropriate. Normality test was performed by Shapiro-Wilk test

^#^P-value < 0.05

**Table 2:** Intraoperative records and managements.

	Total (n = 2576)	TIVA group (n = 1374)	Volatile group (n = 1202)	P-value***
**Intraoperative records**				
**Volatile MAC mean**	0.75 (0.67, 0.82)		0.75 (0.67, 0.82)	
**Desflurane**			**320 (27%)**	
**Sevoflurane**			**882 (73%)**	
**Propofol Ce. mean (mcg/ml)**	3.2 (2.8, 3.5)	3.2 (2.8, 3.5)		
**Remifentanil Ce. mean (ng/ml)**	0.46 (0, 1.03)	0.87 (0.52, 1.39)	0 (0, 0.91)	0.209
**BIS mean**	40 ± 7.1	40 ± 7.11	42 ± 6.7	<0.001^#^
**SpO**_**2**_ **mean (%)**	99 ± 1.9	99 ± 2.0	99 ± 1.7	<0.001^#^
**BT mean (°C)**	35.96 ± 1.14	35.95 ± 1.31	35.99 ± 0.53	0.444
**Blood loss (ml)**	10 (0, 50)	10 (0, 30)	30 (0, 50)	<0.001^#^
**Crystalloid (ml)**	150 (100, 200)	120 (100, 200)	150 (100, 220)	<0.001^#^
**Colloid (ml)**	0 (0, 0)	0 (0, 0)	0 (0, 0)	0.756
**Anesthesthetic duration**	121.16 ± 65.31	104.63 ± 49.7	140.05 ± 75.18	<0.001^#^
**Hemodynamic parameters**				
**Baseline SBP (mmHg)	170 ± 36.1	172 ± 36.1	167 ± 35.9	0.006^#^
**Baseline MBP (mmHg)	116 ± 21.3	1156 ± 20.7	115 ± 22.4	0.528
**Baseline DBP (mmHg)	79.9 ± 17.1	79 ± 16.6	81 ± 18.0	0.151
**Baseline HR (beat/min)	78 ± 15.3	77 ± 15.2	79 ± 15.5	0.033^#^
**SBP mean (mmHg)**	131 ± 20.7	131 ± 21.8	131 ± 19.4	0.675
**SBP sd**	23.73 ± 11.2	22.36 ± 10.3	25.31 ± 11.97	<0.001^#^
**MBP mean (mmHg)**	93 ± 12.9	92 ± 12.7	95 ± 13.0	<0.001^#^
**MBP sd**	15.21 ± 16.83	13.6 ± 7.06	17.04 ± 23.34	<0.001^#^
**DBP mean (mmHg)**	68 ± 11.8	66 ± 11.2	70 ± 12.2	<0.001^#^
**DBP sd**	10.68 ± 4.3	9.57 ± 3.68	11.95 ± 4.61	<0.001^#^
**HR mean (mmHg)**	77 ± 14.7	76 ± 14.3	79 ± 15.1	<0.001^#^
**HR sd**	8.15 ± 4.38	7.64 ± 4.06	9.12 ± 4.8	<0.001^#^
**Hypertensive duration (min)**	100.56 ± 99.21	93.96 ± 96.96	113.46 ± 102.36	<0.001^#^
**Hypotensive duration (min)**	32.49 ± 70.02	28.14 ± 67.68	41.01 ± 96.96	0.008^#^
**Tachycardia duration (min)**	29.85 ± 81.74	22.08 ± 61.56	44.97 ± 109.53	<0.001^#^
**Bradycardia duration (min)**	48.74 ± 81.72	48.15 ± 96.27	49.89 ± 107.40	0.758
**Intraoperative management**				
**Ephedrine**				
**Received (n)**	1084	890	194	0.299
**Mean dose (mg)**	10.73 ± 8.35	10.62 ± 7.55	10.99 ± 10	0.711
**Phenylephrine**				
**Received (n)**	1746	776	970	0.333
**Median dose (mcg)**	200 (100, 300)	200 (100, 312.5)	150 (100, 300)	0.217
**Nicardipine**				
**Received (n)**	74	46	28	0.209
**Mean dose (mg)**	1.16 ± 1.25	1.05 ± 0.89	1.35 ± 1.68	0.380
†**Epinephrine**				
**Received (n)**	12	7	5	0.407
**Mean dose (mcg)**	23.18 ± 29.11	30.3 ± 37.05	13.21 ± 7.64	0.276
**Esmolol**				
**Received (n)**	96	35	61	
**Mean dose (mg)**	22.92 ± 19.5	25 ± 21.73	21.73 ± 18.18	0.455
**Dopamine infusion**				
**Received (n)**	27	19	8	0.824
**Mean dose (mcg/kg/min)**	6.39 ± 65.78	6.83 ± 65.26	5.33 ± 67.14	0.736
**Epinephrine infusion**				
**Received (n)**	30	12	18	0.432
**Mean dose (mcg/kg/min)**	0.12 ± 1.630	0.10 ± 1.520	0.17 ± 1.874	0.517

*Abbreviation: MAC, Minimum alveolar concentration; CE, Effect site concentration; SBP, systolic blood pressure; MBP, mean blood pressure; DBP, diastolic blood pressure; HR, heart rate; sd, standard deviation; BIS, bispectral index score; SpO_2_, oxygen saturation by pulse oximetry; BT, body temperature

**Baseline: preanesthetic value

***P-value for an analysis between TIVA group and volatile group

^†^The volume of epinephrine administered by bolus injection.

^††^All continuous variables are reported as mean ± SD or median (IQR) and all categorical variables as n (proportion, %). Data were analyzed using t-tests or Mann-Whitney U test for continuous variable, chi-square or Fisher’s exact test as appropriate. Normality test was performed by Shapiro-Wilk test.

^#^P-value < 0.05

**Table 3:** Incidence of MACE and the causes.

	Total (n = 2576)	TIVA group (n = 1374)	Volatile group (n = 1202)	P-value**
**MACE**	213 (8.27%)	73 (5.31%)	140 (11.65%)	<0.001^#^
**Cause Category of MACE**				0.089
**• All-cause death**	73 (34.27%)	22 (30.14%)	51 (36.43%)	
**• Cardiac arrest**	2 (0.94%)	2 (2.74%)	0 (0%)	
**• Congestive heart failure**	3 (1.41%)	2 (2.74%)	1 (0.71%)	
**• MI**	21 (9.86%)	6 (8.22%)	15 (10.71%)	
**• Stress-induced cardiomyopathy**	1 (0.47%)	1 (1.37%)	0 (0%)	
**• Cardiac arrhythmia**	4 (1.88%)	1 (1.37%)	3 (2.14%)	
**• Severe hypotension**	104 (48.83%)	37 (50.68%)	67 (47.86%)	
**• Pulmonary edema**	3 (1.41%)	0 (0%)	3 (2.14%)	
**• Pulmonary embolism**	2 (0.94%)	2 (2.74%)	0 (0%)	

*Abbreviations: MACE, major adverse cardiovascular event; MI, myocardial infarction.

** P-value for the analysis between the TIVA and volatile groups

** All variables are reported as n (proportion, %). Data were analyzed using the chi-square or Fisher’s exact test as appropriate.

^#^P-value < 0.05

**Fig 2:**
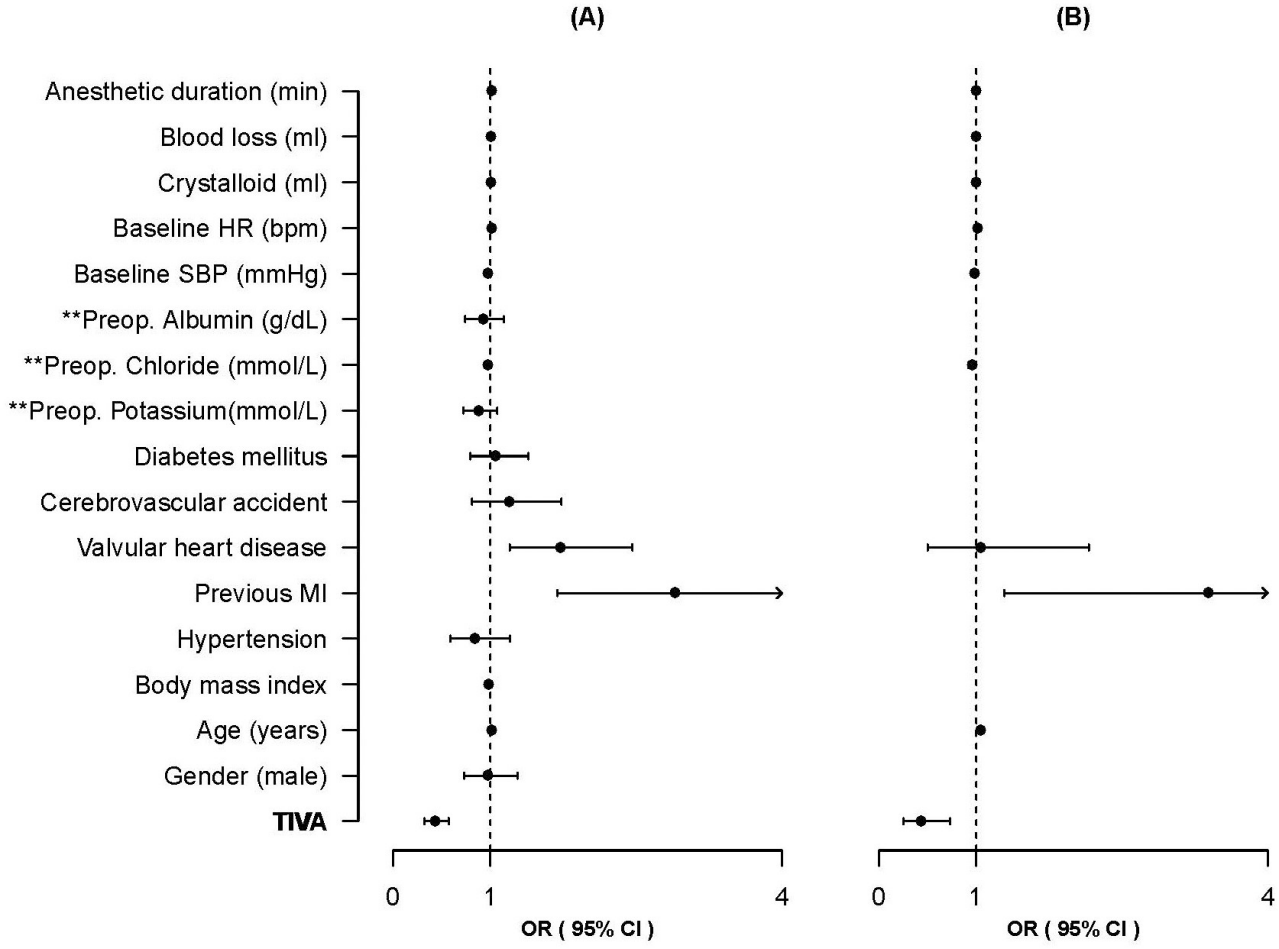
Forest plot. (a) Results of univariate analysis. (b) Only the factors finally selected using the variable selection method were plotted by stepwise selection. TIVA, total intravenous anesthesia; MI, myocardial infarction; Baseline SBP, preanesthetic systolic blood pressure; Baseline HR, preanesthetic heart rate; MBP, mean blood pressure during anesthesia; HR, heart rate during anesthesia. OR (95% CI): odds ratio and 95% confidence interval.

Table 4 presents the logistic regression results. There was no missing co-variate data, we could include all cases for the analysis. Patient characteristics that differed between the groups or are reportedly significantly associated with an increased risk for mortality in ESRD [22–24] were included in the univariate analysis. The calculated duration values, intraoperative mean blood pressures or heart rate were not included because they could be associated with anesthetic method. Only the baseline blood pressure and heart rate were included in the analysis. The multivariate analysis included 10 factors, including the anesthesia method, of which five factors were significant (Table 4). A forest plot (Fig 2) was prepared with only the factors finally selected using the variable selection method with stepwise selection. Factors that were associated with a significantly lower MACE risk included preoperative chloride concentration (OR: 0.96; 95% CI, 0.92–0.99), baseline SBP (OR: 0.98; 95% CI, 0.98–0.99), and propofol TIVA (OR: 0.37; 95% CI, 0.22–0.60) (Fig 2). The OR of propofol TIVA for MACE decreased from 0.43 to 0.37. Patients who were older (OR: 1.04; 95% CI, 1.02–1.06) and those previously diagnosed with MI (OR: 3.26; 95% CI, 1.31–8.11) were at significantly higher risk for MACE.

**Table 4:** Univariate and multivariate analyses.

	Univariable analysis		Multivariable analysis	
	OR (95% CI)	P-value	OR (95% CI)	P-value
**TIVA**	0.43 (0.32–0.57)	<0.001^#^	0.43 (0.25–0.73)	0.002^#^
**Gender (male)**	0.97 (0.73–1.28)	0.808		
**Age (years)**	1.01(1.00–1.03)	0.012^#^	1.04 (1.02–1.07)	<0.001^#^
**Body mass index**	0.98 (0.95–1.01)	0.245		
**Hypertension**	0.84 (0.59–1.20)	0.346		
**Previous MI**	2.90 (1.69–4.95)	<0.001^#^	3.39 (1.29–8.93)	0.014^#^
**Valvular heart disease**	1.72 (1.20–2.46)	0.003^#^	1.04 (0.50–2.16)	0.919
**Cerebrovascular accident**	1.19 (0.81–1.73)	0.375		
**Diabetes mellitus**	1.05 (0.79–1.39)	0.747		
**Preop. Potassium(mmol/L)**	0.88 (0.72–1.07)	0.209		
**Preop. Chloride (mmol/L)**	0.97 (0.95–1.00)	0.063	0.96 (0.92–0.99)	0.022^#^
**Preop. Albumin (g/dL)**	0.92 (0.74–1.14)	0.455		
**Baseline SBP (mmHg)**	0.97 (0.97–0.98)	<0.001^#^	0.98 (0.98–0.99)	<0.001^#^
**Baseline HR (bpm)**	1.01(1.00–1.02)	0.005^#^	1.01(0.99–1.03)	0.238
**Crystalloid (ml)**	1.00 (1.00–1.00)	<0.001^#^	1.00 (1.00–1.00)	0.632
**Blood loss (ml)**	1.00 (1.00–1.00)	0.025^#^	1.00 (1.00–1.00)	0.443
**Anesthetic duration (min)**	1.01 (1.00–1.01)	<0.001^#^	1.00 (1.00–1.01)	0.290

*Abbreviations: TIVA, total intravenous anesthesia; MI, myocardial infarction; Baseline SBP, preanesthetic systolic blood pressure; Baseline HR, preanesthetic heart rate; MBP, mean blood pressure during anesthesia; HR, heart rate during anesthesia; Preop. Preoperative.

**OR (95% CI): odds ratio and 95% confidence interval.

^#^P-value < 0.05

## Discussion

This retrospective study of 2576 cases in ESRD patients evaluated the relationship between the postoperative outcome and the anesthetic method. Propofol TIVA was associated with reduced odds of postoperative MACE after adjusting for confounding variables. We inferred that the anesthetic method affected the postoperative outcome in patients with ESRD and that patients had a better outcome if they received propofol TIVA rather than volatile anesthesia.

We cannot conclude with certainty that the difference in the anesthetic method was a definite cause of the difference in outcome because of the retrospective nature of this study. Moreover, it was not possible to determine the mechanism for this difference. However, the possibility of improving the outcome of TIVA was sufficiently demonstrated in this study.

Surgery causes significant physiological stress and induces oxidative stress through the overproduction of free radicals or reactive oxygen species (ROS) [17, 18]. The overproduction of ROS during surgery damages cellular macromolecules, including DNA, proteins, and lipids. The balance between the oxidative stress from surgery and the antioxidant capacity of the body is believed to contribute to surgical complications [27]. Propofol is a phenolic derivative with the formula 2,6-diisopropylphenol that acts as an antioxidant by reacting with free radicals [17]. Several *in-vivo* and *in-vitro* studies have demonstrated the free radical scavenging properties of propofol either by directly chelating ROS, inhibiting lipid peroxidation, or increasing antioxidant capacity [18, 28–31]. Bellanti et al. reported that propofol reduces mitochondrial dysfunction by preserving respiratory activity, membrane potential and energy homeostasis, and limiting free radicals’ production [9]. These antioxidant activities are significant, fast, stable, and dynamic at clinical concentrations [30, 32]. Therefore, propofol can potentially influence the postoperative outcome through its antioxidant effects and their roles in organ protection.

Other mechanisms that have been linked to the potential organ-protective effect of propofol include anti-apoptosis by suppressing the pro-apoptotic protein Bax and an anti-inflammatory effect by inhibiting macrophage production of tumor necrosis factor-α and interleukins [13, 14]. Perioperative inflammation increases vulnerability to postoperative organ dysfunction and mortality in patients undergoing surgery [33, 34]. Surgery and the stress response suppress the activity of the cell-mediated immune system, particularly natural killer cell activity [35]. Propofol has been reported to be associated with a less adverse inflammatory profile and has immunoprotective effects. It preserves the function of natural killer cells [15], it enhances activation and differentiation of peripheral T-helper cells that augment cellular immunity [16], and it diminishes the production of cytokines [36]. Overall, propofol has been shown to have antioxidant and anti-inflammation properties, and produce a protective effect on the immune system, which may have contributed to the better outcomes after surgery compared with volatile anesthesia.

Because of severe comorbidities and a lack of renal function, patients with ESRD are at considerable perioperative risk. The literature consistently demonstrates considerably higher mortality in ESRD patients than their counterparts with normal renal function during the perioperative period of cardiac and noncardiac surgeries [37–39]. In previous studies, the overall mortality rate for ESRD patients undergoing general surgery was approximately 4% [40, 41]. This is similar with our volatile group mortality (4.2%, 51 death among 1202) but lower in TIVA group (1.6%, 22 death among 1374). Several studies have found that the high morbidity and mortality of ESRD patients is primarily due to cardiovascular complications [41–43]. Cardiovascular causes, including arrhythmias, cardiac arrest, congestive heart failure, acute MI, and atherosclerotic heart disease are responsible for 48% of deaths among dialysis patients [2]. Additionally, postoperative adverse cardiac events are a major cause of morbidity and mortality in patients after non-cardiac surgery in general population [44, 45]. Therefore, we evaluated postoperative MACE in ESRD patients after non-cardiac surgery, the most important outcome, to investigate the safety of propofol TIVA in ESRD patients. The adjusted OR of propofol TIVA for postoperative MACE was 0.35, which was lower than the unadjusted OR of 0.43 when compared to volatile anesthesia in patients with ESRD. We speculate that the previously described beneficial effects of propofol were maximized in this vulnerable patient group.

As introduced above, theoretical advantages of propofol TIVA are anticipated for ESRD patients. However, the complexity and variability in drug handling may remain an issue for using TIVA and may restrict the use of propofol TIVA in ESRD patients. In 1998, Ickx et al. investigated the pharmacokinetics and pharmacodynamics of propofol in 11 patients with ESRD compared with healthy patients [46]. They found that ESRD requiring hemodialysis did not significantly affect the pharmacokinetics of propofol. The mean total body clearance of propofol was not reduced significantly in the ESRD group (30.66 mL∙kg^-1^∙min^-1^) compared with the control group (33.75 mL∙kg^-1^∙min^-1^). ESRD patients tended to exhibit a greater volume distribution at steady state than patients in the control group (11.25 vs. 5.79 mL∙kg^-1^, respectively). Ickx et al. also found that elimination half-life values were unchanged by renal failure. Mean times to induction of anesthesia were similar in both groups, and recovery time was significantly shorter in the ESRD group than in the control group. They concluded that the pharmacokinetic and pharmacodynamic profiles of propofol after infusion were not markedly affected by renal failure. Dahaba et al. reported that ESRD does not prolong recovery from TIVA with remifentanil and propofol and concluded that patients with ESRD can be anesthetized with TIVA using propofol [47]. Together, these previous findings indicate that TIVA can be used in ESRD patients, but no convincing data were available to show that the outcome was improved by TIVA compared to volatile anesthesia. Although many studies have found that propofol-based TIVA may contribute to a better surgical outcome [13–19], ESRD patients are excluded in most studies. The ESRD patients are very susceptible to the adverse effects of surgery primarily due to cardiovascular complications [41–43]. Therefore, it would be more important in ESRD patients. Our findings confirmed that propofol TIVA was associated with a decreased risk of postoperative MACE in ESRD patients who are at a higher risk of postoperative MACE. These results highlight the potential utility of TIVA for postoperative safety in ESRD patients.

Hypotension is another reason for reservations using TIVA in ESRD patients. These patients may arrive in the operating room in a volume-depleted state after recent dialysis and fluid restrictions. Thus, they are prone to hypotensive responses to vasodilating drugs with negative inotropic effects. Tailoring blood pressure is very important for ESRD patients to reduce perioperative morbidity. Dahaba et al. demonstrated that TIVA produces more hypotension in ESRD patients than in control subjects and requires more ephedrine, despite a lower concentration of remifentanil [47]. In our study population, MBP and SBP were lower in the TIVA group than the volatile group. This finding is consistent with those of Dahaba et al., but we found that remifentanil concentration was high and did not require more ephedrine or phenylephrine or an inotropic agent in the TIVA group. We also found that the hypotensive duration was longer in the volatile group. Unfortunately, we could not include the hypotensive duration values for analysis, because they may be closely related to anesthetic method difference.

### Limitations

Our study had several limitations. First, this was a retrospective analysis for which the greatest criticism is that baseline characteristics of the two groups differed. The choice of anesthetic was based on the anesthesiologist’s preference, but more patients in the volatile group were female, and more had MI, VHD, and cerebrovascular accident. And although the surgical severity was comparable, there were differences in some surgery types including aortic and other major vascular surgery. Other problems are the volume of blood loss and the volume of infused crystalloid were different between the groups. As has already been shown in many studies, bleeding has a significant effect on patient prognosis. Also, in ESRD, volume infusion can be a burden on the patient. We performed a multivariate analysis to exclude the effects of these differences. However, we could not include all confounding factors such as postoperative pain and pre-and post-operative inflammatory parameters, although these are an influencing factor of postoperative outcomes. Second, hemodialysis-dependent duration was lacking in our study. It may be argued that there is a time-dependent relationship between hemodialysis-dependent duration and outcome. We were unable to determine the hemodialysis duration for each patient due to a lack of formally recorded data. However, it would have not influenced the anesthetic choice. Third, we analyzed based on the surgery case and included the patients who had multiple procedures during the study period. And it is not clear how this influences the outcome. Fourth, improvements in ESRD care over the period could have influenced the outcomes, but the study period was relatively short (2 years) and the proportions of TIVA and volatile agents remained remarkably similar throughout this study. Fifth, if the patients experienced MACE out of the hospital, it could not be included in the analysis. Finally, propofol was used to induce general anesthesia in both groups. However, the dose of propofol is reportedly related to the outcome [12]. We excluded patients who received both forms of anesthesia within the study period.

In conclusion, after adjusting for established prognostic factors in multivariate models, TIVA remained an independent factor for decreasing postoperative MACE in ESRD patients. These results indicate that propofol TIVA might be a suitable anesthetic method for ESRD patients. However, further studies are needed to conclude this, because this is a retrospective study and assessed in peripheral vascular surgeries, there are limitations to applying to other situations.

## Supporting information

S1 Table Surgery type, severity and postoperative pain. (DOCX)
