# Supplementary material for: The influence of propofol-based total intravenous anesthesia on postoperative outcomes in end-stage renal disease patients: A retrospective observation study
Source: PLoS One. 2021 Jul 22;16(7):e0254014. doi: 10.1371/journal.pone.0254014 (PMC8297880; doi:10.1371/journal.pone.0254014)
Supplement: S1 Table — (DOCX) [file pone.0254014.s001.docx]

**The association of propofol-based total intravenous anesthesia on postoperative outcomes in end-stage renal disease patients: a retrospective observation study**

Ho Bum Cho, M.D., Mun Gyu Kim, M.D., Sun Young Park, M.D., Ph.D., Sanghoon Song, M.D., Youn Sil Jang, M.D., Suyeon Park Ph.D., Hyun Keun Lee, M.D., Jae Hwa Yoo, M.D., Ph.D., Ji Won Chung, M.D., Ph.D., Sang Ho Kim, M.D., Ph.D.

**Table of Contents**

1. **Supplementary Table ………………………………………….………….……2**

**S1 Table. Surgery type, severity and Postoperative pain**

|  | **Total**  **(n=2576)** | **TIVA group**  **(n=1374)** | **Volatile group**  **(n=1202)** | **P-value**** | |
| --- | --- | --- | --- | --- | --- |
| **Surgery type** |  |  |  | NA | |
| **- Aortic and other major vascular surgery** | 21 (0.81%) | 6 (0.44%) | 15 (1.25%) |  | |
| **- Breast surgery** | 5 (0.19%) | 0 (0%) | 5 (0.42%) | |  |
| **- Endoscopic procedures** | 23 (0.89%) | 13 (0.95%) | 10 (0.83%) |  | |
| **- Head & neck surgery** | 43 (1.67%) | 24 (1.75%) | 19 (1.58%) |  | |
| **- Intraperitoneal and intrathoracic surgery** | 21 (0.82%) | 12 (0.87%) | 9 (0.75%) |  | |
| **- Orthopedic surgery** | 64 (2.48%) | 32 (2.33%) | 32 (2.66%) |  | |
| **- Peripheral vascular surgery** | 2358 (91.54%) | 1266 (92.14%) | 1092 (90.85%) |  | |
| **- Superficial procedure** | 41 (1.59%) | 21 (1.53%) | 20 (1.66%) |  | |
| **Surgery severity** |  |  |  | 0.788 | |
| **- High** | 2379 (92.35%) | 1272 (92.58%) | 1107 (92.1%) |  | |
| **- Intermediate** | 128 (4.97%) | 68 (4.95%) | 60 (4.99%) |  | |
| **- Low** | 69 (2.68%) | 34 (2.47%) | 35 (2.91%) |  | |
| **Postoperative pain** |  |  |  |  | |
| **- PCA** | 405 (16.07%) | 233 (16.97%) | 172 (14.36%) | 0.127 | |
| **- NRS** | 0 (0, 2)  (N=1699) | 1 (0, 2)  (N=1114) | 0 (0, 2)  (N=585) | <0.001^#^ | |

*Abbreviation: PCA, Patient controlled analgesia; NRS, numeric rating scale of pain

** P-value for an analysis between TIVA group and volatile group

***All variables are reported as n (proportion, %) or median (IQR). Data were analyzed using chi-squared test for categorical variable, Mann-Whitney U test for continuous variable.

# P-value < 0.05
